# Robust and accurate prediction of protein–protein interactions by exploiting evolutionary information

**DOI:** 10.1038/s41598-021-96265-z

**Published:** 2021-08-19

**Authors:** Yang Li, Zheng Wang, Li-Ping Li, Zhu-Hong You, Wen-Zhun Huang, Xin-Ke Zhan, Yan-Bin Wang

**Affiliations:** 1 School of Information Engineering, Xijing University, Xi’an, 710123 China; 2 School of Cyber Science and Technology, Zhejiang University, Hangzhou, 310027 China

**Keywords:** Bioinformatics, Proteomics

## Abstract

Various biochemical functions of organisms are performed by protein–protein interactions (PPIs). Therefore, recognition of protein–protein interactions is very important for understanding most life activities, such as DNA replication and transcription, protein synthesis and secretion, signal transduction and metabolism. Although high-throughput technology makes it possible to generate large-scale PPIs data, it requires expensive cost of both time and labor, and leave a risk of high false positive rate. In order to formulate a more ingenious solution, biology community is looking for computational methods to quickly and efficiently discover massive protein interaction data. In this paper, we propose a computational method for predicting PPIs based on a fresh idea of combining orthogonal locality preserving projections (OLPP) and rotation forest (RoF) models, using protein sequence information. Specifically, the protein sequence is first converted into position-specific scoring matrices (PSSMs) containing protein evolutionary information by using the Position-Specific Iterated Basic Local Alignment Search Tool (PSI-BLAST). Then we characterize a protein as a fixed length feature vector by applying OLPP to PSSMs. Finally, we train an RoF classifier for the purpose of identifying non-interacting and interacting protein pairs. The proposed method yielded a significantly better results than existing methods, with 90.07% and 96.09% prediction accuracy on *Yeast* and *Human* datasets. Our experiment show the proposed method can serve as a useful tool to accelerate the process of solving key problems in proteomics.

## Introduction

Proteins are the main functional components of biological cells, and they usually interact with DNA or other proteins in a specific way to perform their functions. Protein–protein interactions (PPIs) are critical to understanding the function of proteins and further manipulating many biological processes^1^. Therefore, the analysis of protein interactions has gradually become a hot topic in proteomics research. Thus far, researchers have discovered various experimental methods for detecting large-scale PPIs, including yeast two-hybrid^2,3^, protein chips^4^, tandem affinity purification^5^, immunoprecipitation^6^, and other high-throughput biotechnology. The rapid development of these high-throughput technologies has also accumulated available experimental data for the study of protein–protein interactions^7^. Nevertheless, biological experimental methods are expensive, time consuming, and labor intensive. Moreover, these methods typically perform poorly and are prone to produce low rates of true negative and true positive predictions^8–10^. Thus, an effective computational method to predict PPIs is highly desirable, and it may also alleviate the bottleneck of experimental methods^11,12^.

Currently, many computational methods based on various data types have been developed for predicting protein–protein interactions. The data sources involved in these methods mainly include literature mining knowledge^13^, gene fusion^14^, phylogenetic profiles^15^, gene ontology annotations^16^, gene neighborhood^17^, and co-evolution analysis of interacting proteins^18^. However, these methods are not commonly used to predict PPIs as they are difficult to apply if a priori information about the protein is not available. Moreover, the rapid development of genomic technology has led to an excessive accumulation of protein sequence data. Hence, it is very popular to predict protein–protein interactions based on protein sequence information^19,20^.

Numerous previous studies have found that PPIs can be detected using only protein amino acid sequence data^21,22^. Guo et al.^23^ reported a sequence-based method that combines auto-covariance (AC) and support vector machine (SVM) to predict PPIs. Among them, AC considers the neighbouring effect and explains the interaction between a certain number of residues in the sequence. The accuracy of this method on the *Saccharomyces cerevisiae* data was 88.09%. Pitre et al.^24^ developed a computational engine called PIPE to predict protein–protein interactions. The engine can efficiently detect interactions among yeast protein pairs. The experimental results show that the PIPE algorithm achieves a sensitivity of 61% with 89% specificity and an average accuracy of 75% on yeast dataset. You et al.^25^ proposed a hierarchical PCA-EELM method to predict PPIs, which utilizes only protein sequence information. Lei et al.^26^ showed a neighbor affinity-based core-attachment method (NABCAM) to predict protein complexes from dynamic PPI networks. Huang et al.^19^ presented a sequence-based substitution matrix representation (SMR) method to predict PPIs by using discrete cosine transform (DCT). This method yielded an average accuracy of 96.28% on the yeast dataset. Ding et al.^27^ proposed a matrix-based protein sequence representation method that combines HOG and SVD feature representations as well as random forest classifiers to predict PPIs. Wang et al.^28^ presented a computational model to predict PPIs, which is based on a Zernike moment (ZM) feature descriptor and a probabilistic classification vector machine (PCVM) algorithm. Although the existing prediction methods for protein–protein interactions have been developed by many investigators, there is still room for improvement in algorithms and prediction accuracy of PPIs.

In this paper, we report a protein sequence-based approach to detect protein–protein interactions. Specifically, all protein sequences were first converted to a position-specific scoring matrix (PSSM). Then, we use the orthogonal locality preserving projections (OLPP) algorithm to extract feature mathematical descriptors from each protein PSSM to obtain more representative information. Finally, we use the ensemble learning method in machine learning to perform the classification tasks of PPIs. The proposed method was applied to highly trusted *Yeast* and *Human* datasets to test the performance of PPIs prediction models. In addition, we demonstrate the predictive power of the proposed model on four separate datasets including *C. elegans*, *H. pylori*, *H. sapiens,* and *M. musculus*. Through further comparative experiments, our method obtains good prediction accuracy, which can reflect the reliability of the proposed method in predicting PPIs.

## Results and discussion

### Evaluation measures

**Figure 1:**
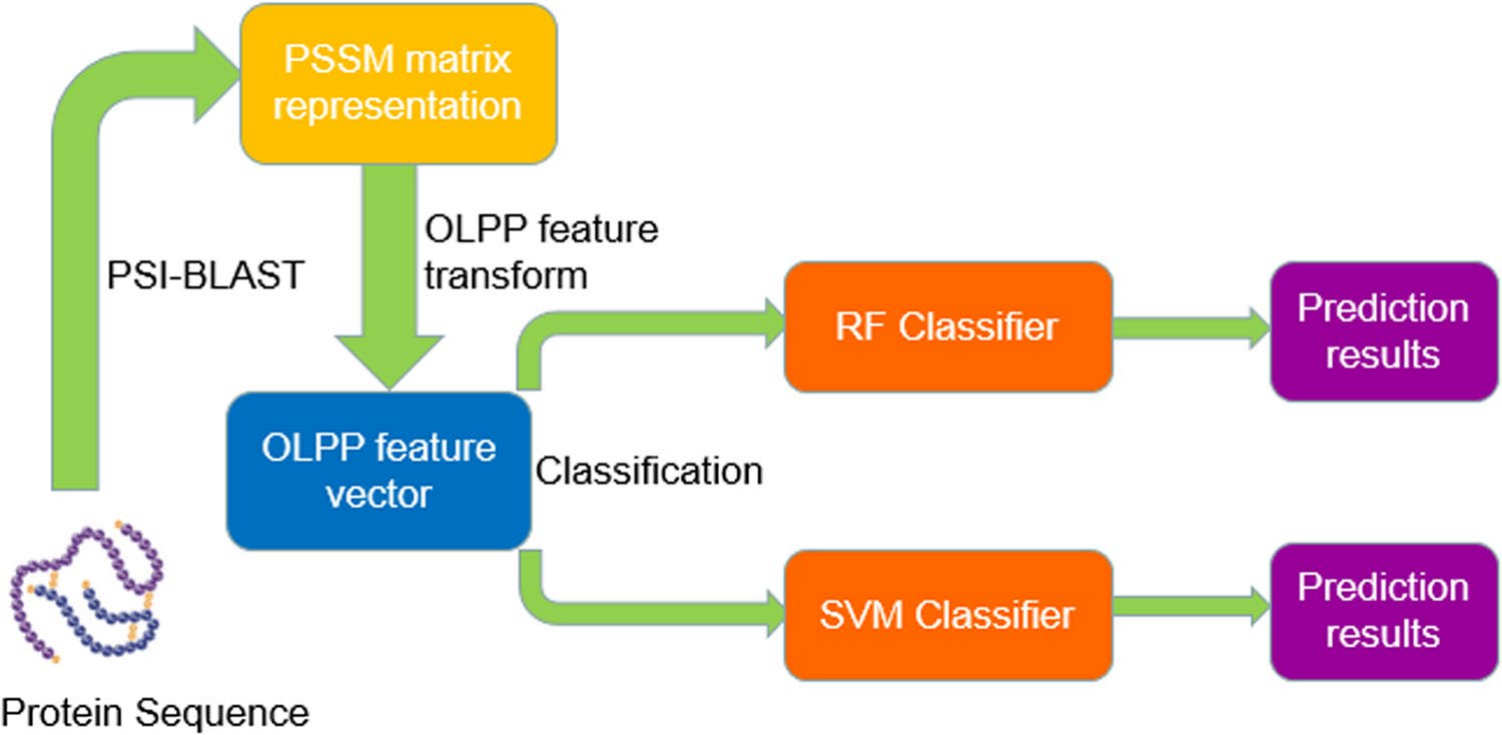
The workflow of the proposed method.

To validate the proposed model, we consider the following evaluation criteria in this experiment. The calculation formulas for overall prediction accuracy (Acc), precision (Pre), sensitivity (Sen), and Matthews correlation coefficient (MCC) are defined as:

1 $$Accuracy = \frac{TN + TP}{{TN + TP + FN + FP}},$$

2 $$Precision = \frac{TP}{{FP + TP}},$$

3 $$Sensitivity = \frac{TP}{{TP + FN}},$$

4 $$MCC = \frac{(TP \times TN) - (FP \times FN)}{{\sqrt {{(}TP + FP{)} \times {(}TN + FN{)} \times {(}TN + FP{)} \times {(}TP + FN{)}} }},$$

where $TN$ is the number of true negatives, indicating that the non-interacting proteins are predicted correctly; $TP$ is the amount of true positives, representing that the interacting proteins are predicted correctly; $FN$ is the number of false negatives, indicating that the interacting proteins are predicted to be non-interacting; and $FP$ is the amount of false positives, representing that the non-interacting proteins are predicted to have interaction. Additionally, the receiver operating characteristic (ROC)^29^ curves and the area under the ROC curve (AUC)^30^ were also calculated to further evaluate the discriminatory accuracy of the proposed model. The workflow of the proposed method is shown in Fig. 1.

### Assessment of prediction

**Figure 2:**
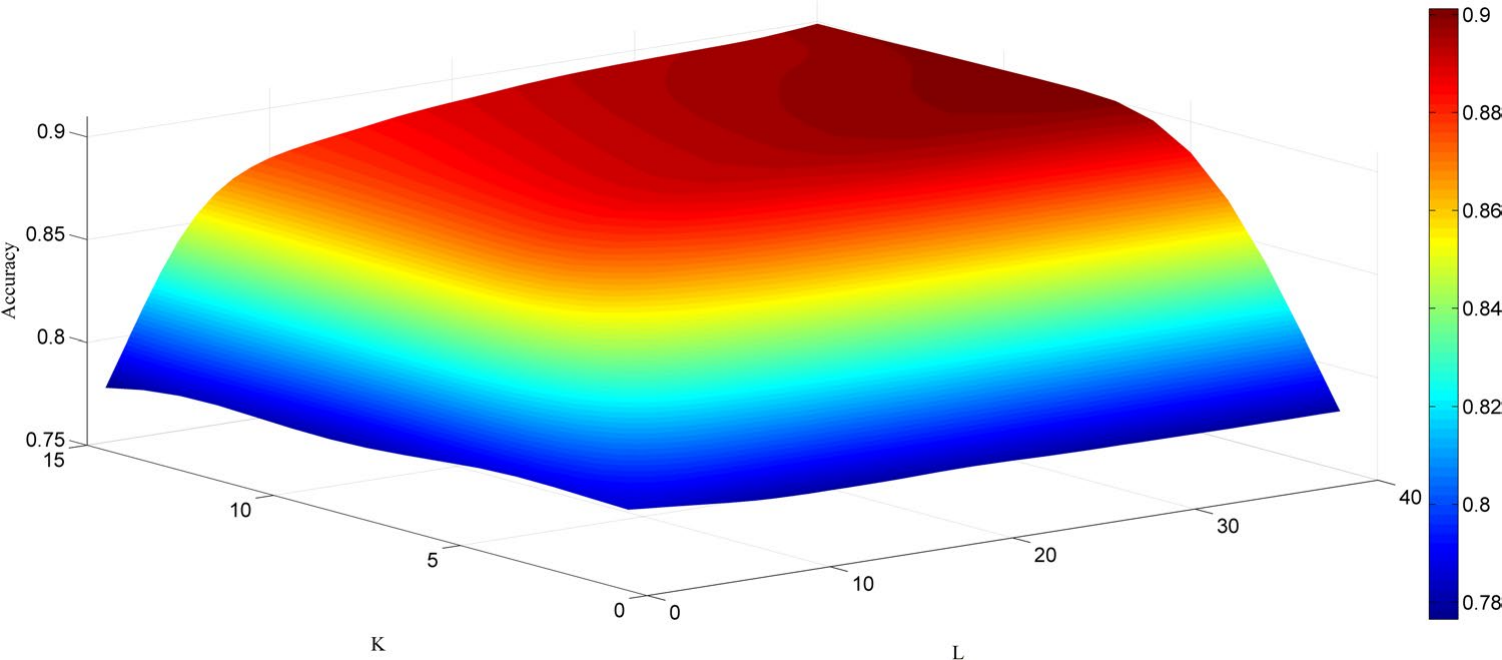
The accuracy surface obtained from the RoF algorithm for optimizing parameters *K* and *L*.

We applied the proposed method to two popular PPIs datasets to verify the performance of the model, including *Yeast* and *Human* datasets. In addition, to avoid over-fitting problems in the experiment, we used a five-fold cross-validation method to evaluate prediction performance. Specifically, we divided the entire dataset into five parts, four of which were used for training and one part was used for testing. In this way, we can obtain five separate models from the *Yeast* and *Human* datasets and perform five independent experiments. To be fair, we set the same parameters for the rotation forest classifier on different datasets. In this experiment, we use a grid search method to optimize two important parameters of the RoF algorithm. Figure 2 presents the prediction results of the RoF algorithm under different parameters. Here, the parameter $K$ (the amount of feature subsets) is set to 10 and the parameter $L$ (the amount of decision trees) is set to 35. The predicted results obtained by combining the proposed model with the five-fold cross-validation method on different datasets are shown in Table 1.

**Table 1:** Five-fold cross-validation prediction results using the proposed method on two dataset.

Data sets	Acc (%)	Pre (%)	Sen (%)	MCC (%)
*Yeast*	90.07 ± 0.60	90.24 ± 0.56	89.83 ± 1.41	82.10 ± 0.97
*Human*	96.09 ± 0.24	96.56 ± 0.36	95.20 ± 0.34	92.47 ± 0.46

**Figure 3:**
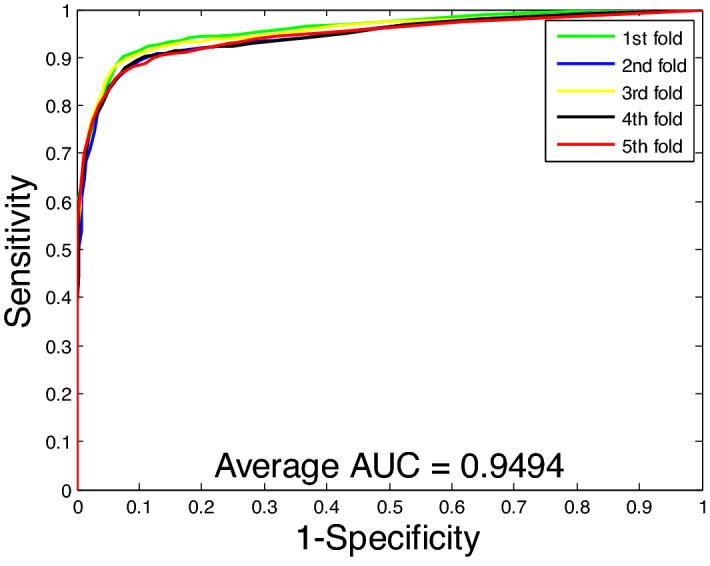
ROC curves performed using the proposed method on *Yeast* dataset.

**Figure 4:**
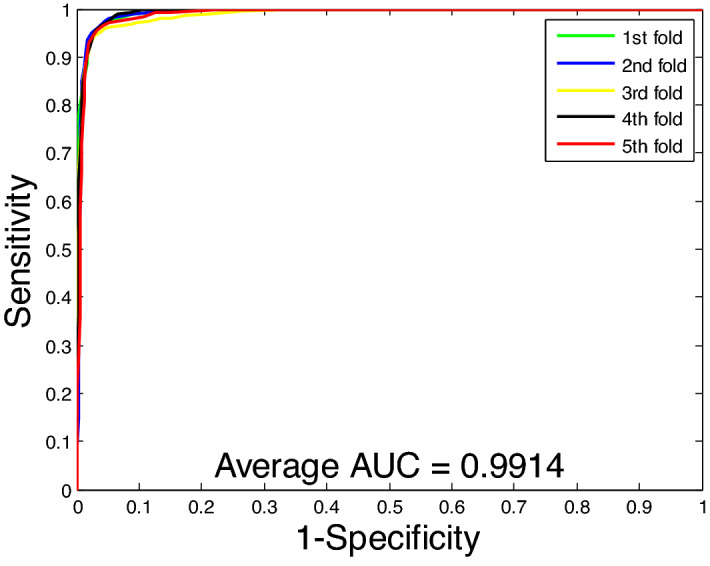
ROC curves performed using the proposed method on *Human* dataset.

From Table 1, we can see that the proposed method for predicting PPIs has a good performance on the *Yeast* dataset. Its average accuracy, precision, sensitivity, and MCC were 90.07%, 90.24%, 89.83%, and 82.10%, respectively, and their standard deviations were 0.60%, 0.56%, 1.41%, and 0.97%, respectively. In addition, our method also achieved satisfactory results on the *Human* dataset. Its average accuracy, precision, sensitivity, and MCC were 96.09%, 96.56%, 95.20%, and 92.47%, respectively, and the standard deviations were 0.24%, 0.36%, 0.34%, and 0.46%, respectively. Figures 3 and 4 show the ROC curves of the proposed method on these two datasets, respectively. In the figure, the Y-axis refers to the true positive rate (TPR) and the X-axis refers to the false positive rate (FPR). To further evaluate the performance of the RoF classifier, we also obtained average AUC values of 94.94% and 99.14% on the *Yeast* and *Human* datasets, respectively. Observing these results, our method can achieve higher accuracy and lower standard deviation. This further indicates that the proposed method can effectively detect PPIs.

### Comparison of proposed method and support vector machine method

Many algorithms and knowledge about machine learning are used to detect PPIs. Among them, support vector machine (SVM) is a popular supervised learning algorithm. To evaluate the predictive ability of the proposed model, we used the same feature extraction method to compare the prediction results of the two classifiers including RoF and SVM on *Yeast* and *Human* datasets. In this experiment, we use the LIBSVM tools as an SVM classifier, which can be downloaded from https://www.csie.ntu.edu.tw/~cjlin/libsvm/. To improve the prediction results of the SVM classifier on these two datasets, we use a grid search method to select two important parameters of SVM, namely the regularization parameter c and the kernel parameter g. When predicting PPIs on the *Yeast* dataset, the parameters c and g are set to 4 and 1, respectively. When detecting PPIs on the *Human* dataset, the parameters c and g are set to 8 and 1, respectively. Furthermore, we chose the radial basis function as the kernel function in this experiment.

**Figure 5:**
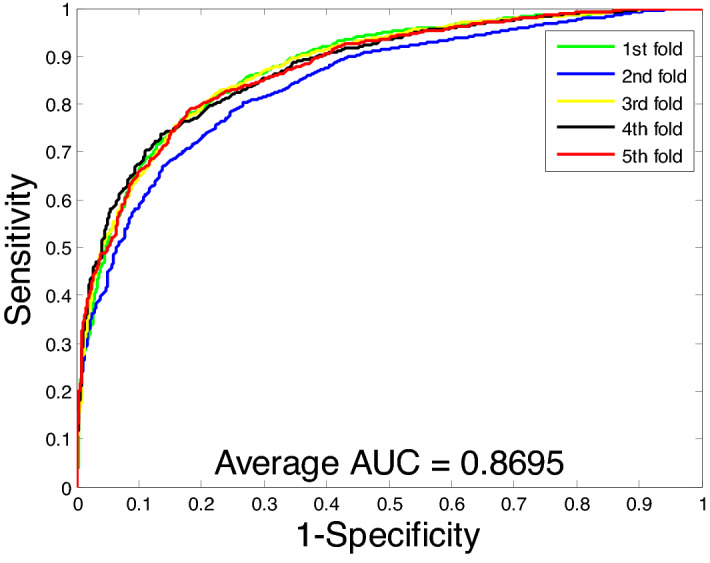
ROC curves performed using the SVM method on *Yeast* dataset.

**Figure 6:**
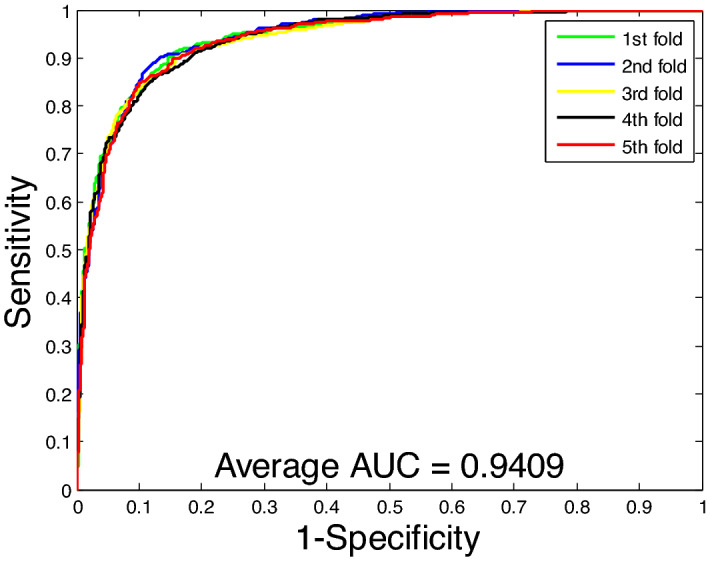
ROC curves performed using the SVM method on *Human* dataset.

From Table 2, we can observe that the SVM-based method achieves an average accuracy of 78.96%, an average precision of 79.08%, an average sensitivity of 78.76%, and an average MCC of 66.80% by using fivefold cross-validation on the *Yeast* dataset. However, the RoF-based methods achieved average accuracy, precision, sensitivity, and MCC of 90.07%, 90.24%, 89.83%, and 82.10%, respectively. At the same time, we also compared the prediction results of the two classifiers on the *Human* dataset using the same feature extraction method. Similarly, we can see that the SVM-based classifier has 87.23% average accuracy, 87.23% average precision, 85.83% average sensitivity, and 77.66% average MCC on the *Human* dataset. In addition, we plot the ROC curves on the two datasets based on the SVM model and calculate the average AUC as shown in Figs. 5 and 6. By comparing these experimental data, we can see that RoF classifiers are significantly better than SVM classifiers in predicting PPIs.

**Table 2:** Comparison of the results of the proposed method and SVM by using five-fold cross-validation on two datasets.

Data sets	Classifier	Acc (%)	Pre (%)	Sen (%)	MCC (%)
*Yeast*	SVM	78.96 ± 1.55	79.08 ± 1.03	78.76 ± 2.37	66.80 ± 1.75
	RoF	90.07 ± 0.60	90.24 ± 0.56	89.83 ± 1.41	82.10 ± 0.97
*Human*	SVM	87.23 ± 0.57	87.23 ± 0.58	85.83 ± 1.16	77.66 ± 0.87
	RoF	96.09 ± 0.24	96.56 ± 0.36	95.20 ± 0.34	92.47 ± 0.46

### Comparison time performance with SVM-based method.

In this section, we compare the training time required by RoF and SVM algorithms on two datasets, by using the same OLPP feature extraction method on the same machine configuration. Table 3 gives the comparison results of the training time required by different algorithms on the *Yeast* and *Human* datasets. It can be shown that the training time of OLPP + RoF method is 401 s higher than that of OLPP + SVM method and the accuracy is improved by about 10% on the *Yeast* dataset. Similarly, the training time of OLPP + RoF method is 170 s while the training time of OLPP + SVM method is 110 s on the *Human* dataset. Although the training speed of the latter is 60 s faster than that of the former, the accuracy is reduced by about 9%. As a result, the RoF algorithm is superior to the SVM algorithm in terms of both prediction accuracy and training time.

**Table 3:** Comparison time performance with SVM-based methods.

Data sets	Method	Time (s)/fold	Accuracy (%)
*Yeast*	OLPP + RoF	237	90.21
	OLPP + SVM	638	79.53
*Human*	OLPP + RoF	170	96.69
	OLPP + SVM	110	87.25

### Comparison with other methods

Thus far, many computational methods have been developed to detect PPIs. In particular, machine learning algorithms have also received widespread attention from researchers. In this section, we compare the proposed method with the currently known methods to further evaluate the predictive power of the model. Tables 4 and 5 summarize the predicted results of other existing methods on *Yeast* and *Human* datasets, respectively. From Table 4, we can see that the accuracy of the proposed method is 90.07%, the sensitivity is 89.83%, the precision is 90.24% and the MCC is 82.10% with the corresponding standard deviations of 0.60, 1.41, 0.56, and 0.97, respectively on the *Yeast* dataset. Similarly, we can find the prediction results of different methods on the *Human* dataset from Table 5. The average accuracy of the proposed method for PPIs prediction reached 96.09%, the sensitivity reached 95.20% and the MCC reached 92.47%. Comparing these results, we can find that the proposed method is a stable and reliable model for predicting PPIs.

**Table 4:** Performance comparisons of 12 methods on the *Yeast* dataset.

Method	Feature	Classifier	Acc (%)	Sen (%)	Pre (%)	MCC (%)
Du et al.^a^	Mutiple	DL	94.43 ± 0.30	92.06 ± 0.36	96.65 ± 0.59	88.97 ± 0.62
Wong et al.^b^	PR-LPQ	RoF	93.92 ± 0.36	91.10 ± 0.31	96.45 ± 0.45	88.56 ± 0.63
Wang et al.^c^	Bio2Vec	CNN	93.30	92.70	93.55	87.49
You et al.^d^	MCD	SVM	91.36 ± 0.36	90.67 ± 0.69	91.94 ± 0.62	84.21 ± 0.59
An et al.^e^	PSSMMF	SVM	90.48 ± 0.76	90.26 ± 0.87	90.58 ± 0.98	82.84 ± 1.27
Wang et al.^f^	3-mers	CNN	90.26	88.14	91.65	82.38
Our method	OLPP	RoF	90.07 ± 0.60	89.83 ± 1.41	90.24 ± 0.56	82.10 ± 0.97
Guo et al.^g^	ACC	SVM	89.33 ± 2.67	89.93 ± 3.68	88.87 ± 6.16	N/A
Zhou et al.^h^	LD	SVM	88.56 ± 0.33	87.37 ± 0.22	89.50 ± 0.60	77.15 ± 0.68
Guo et al.^i^	AC	SVM	87.36 ± 1.38	87.30 ± 4.68	87.82 ± 4.33	N/A
You et al.^j^	Mutiple	PCA-EELM	87.00 ± 0.29	86.15 ± 0.43	87.59 ± 0.32	77.36 ± 0.44
Yang et al.^k^	LD	KNN	86.15 ± 1.17	81.03 ± 1.74	90.24 ± 1.34	N/A

*N/A* means not available. The values behind ± represent the standard deviation.

^a^Mutiple: Multiple Features; DL: Deep Learning; results reported by Ref.^31^.

^b^PR-LPQ: property response matrix-Local Phase Quantization; RoF: Rotation Forest; results reported by Ref.^32^.

^c^Bio2Vec: bio-to-vector; CNN: convolution neural network; results reported by Ref.^33^.

^d^MCD: Multi-scale Continuous and Discontinuous; SVM: Support Vector Machine; results reported by Ref.^34^.

^e^PSSMMF: position-specific scoring matrix with multifeatures fusion; results reported by Ref.^35^.

^f^3-mers: represent a segmentation strategy of sequence; results reported by Ref.^33^.

^g^ACC: Auto Cross Covariance; results reported by Ref.^23^.

^h^LD: Local Description; results reported by Ref.^36^.

^i^AC: Auto Covariance; results reported by Ref.^23^.

^j^PCA-EELM: Principal Component Analysis-Ensemble Extreme Learning Machine; results reported by Ref.^25^.

^k^LD: Local descriptors; KNN: k-nearest neighbors; results reported by Ref.^37^.

**Table 5:** Performance comparisons of 12 methods on the *Human* dataset.

Method	Feature	Classifier	Acc (%)	Sen (%)	Pre (%)	MCC (%)
Du et al.^a^	Mutiple	DL	98.14	96.95	99.13	96.29
Ding et al.^b^	MMI + NMBAC	RF	97.56	96.57	98.30	95.13
Pan et al.^c^	LDA	RF	96.4	94.2	N/A	92.8
Huang et al.^d^	DTC + SMR	WSRC	96.30	92.63	99.59	92.82
Our method	OLPP	RoF	96.09	95.20	96.56	92.47
Ding et al.^e^	MMI	RF	96.08	95.05	96.97	92.17
Pan et al.^f^	LDA	RoF	95.7	97.6	N/A	91.8
Ding et al.^g^	NMBAC	RF	95.59	94.06	96.94	91.21
Pan et al.^h^	AC	RF	95.5	94.0	N/A	91.4
Pan et al.^i^	AC	RoF	95.1	93.3	N/A	91.0
Pan et al.^j^	LDA	SVM	90.7	89.7	N/A	81.3
Pan et al.^k^	AC	SVM	89.3	94.0	N/A	79.2

*N/A* means not available. The values behind ± represent the standard deviation.

^a^Mutiple: Multiple Features; DL: Deep Learning; results reported by Ref.^31^.

^b^MMI + NMBAC: multivariate mutual information + normalized Moreau-Broto Autocorrelation; RF: Random Forest; results reported by Ref.^38^.

^c^LDA: latent dirichlet allocation; RF: Random Forest; results reported by Ref.^39^.

^d^DTC + SMR: discrete cosine transform + substitution matrix representation; WSRC: weighted sparse representation based classifier; results reported by Ref.^19^.

^e^MMI: multivariate mutual information; results reported by Ref.^38^.

^f^LDA: latent dirichlet allocation; RoF: Rotation Forest; results reported by Ref.^39^.

^g^NMBAC: normalized Moreau-Broto Autocorrelation; results reported by Ref.^38^.

^h^AC: auto covariance; results reported by Ref.^39^.

^i^RoF: Rotation Forest; results reported by Ref.^39^.

^j^SVM: Support Vector Machine; results reported by Ref.^39^.

^k^Results reported by Ref.^39^.

### Performance on independent dataset

Although the proposed model has achieved good performance on *Yeast* and *Human* datasets, the suitability of the proposed method for different datasets still needs to be verified. Therefore, we also performed additional experiments to further determine the predictive performance of this model for other species. It should be noticed that there is a biological hypothesis that PPIs are mapped from one species to another. This hypothesis is that many physically interacting proteins have coevolved in a given organism so that they are also likely to interact with proteins from other organisms. In this experiment, we used all of the 11,188 protein pairs of *Yeast* datasets to construct a training set through the previously proposed method. Then, we use four independent datasets as test sets to detect the final prediction model separately. Among them, the four independent test sets are *C. elegans*, *H. pylori*, *H. sapiens,* and *M. musculus* collected in the DIP database. The number of their protein pairs is 4013, 1420, 1412 and 313, respectively. Table 6 shows the PPIs prediction results of the five methods on four species. We can conclude that the proposed model achieved up to 90% prediction accuracy on four independent datasets *C. elegans*, *H. pylori*, *H. sapiens,* and *M. musculus*, which were 90.93%, 92.54%, 92.21%, and 91.37%, respectively. These results not only indicate the outstanding performance of the proposed method in predicting the interaction of other species but also show that the method has good generalization.

**Table 6:** Performance comparisons on four species.

Species	Test pairs	Our Method	Huang et al.^19^	Ding et al.^40^	Wang et al.^41^	Zhan et al.^42^
*C. elegans*	4013	90.93%	81.19%	86.72%	92.60%	93.20%
*H. pylori*	1420	92.54%	82.18%	90.34%	N/A	91.34%
*H. sapiens*	1412	92.21%	82.22%	90.23%	80.10%	91.93%
*M. musculus*	313	91.37%	79.87%	91.37%	89.14%	94.89%

*N/A* means not available.

## Conclusions

Machine learning algorithms play a crucial role in proteomics research as they can quickly and accurately improve the prediction accuracy of PPIs. In this work, we propose an ensemble learning approach to detect PPIs from protein sequences. Orthogonal locality preserving projections are used to extract discriminative features from the PSSM, which can effectively preserve evolutionary information of the protein sequence. Finally, we use a rotation forest model to predict PPIs. To evaluate the reliability of the proposed method for PPIs prediction, we performed experiments on *Yeast* and *Human* datasets to verify the performance of the method. At the same time, we also compared the proposed model with the SVM classifier and other existing models. The experimental results show that our method has achieved good performance in predicting protein interactions and it can be a useful tool for detecting PPIs.

## Materials and methodology

### Data sources

Previous studies have generated many databases of protein–protein interactions, such as Biomolecular Interaction Network Database (BIND)^43^, Molecular Interaction Database (MINT)^44^, and Database of Interacting Proteins (DIP)^45^. To demonstrate the efficacy of the proposed method, we used two publicly available and highly reliable datasets for this study, including *Yeast* and *Human*, which were derived from the database of interacting proteins (DIP) and collected by Guo et al.^23^ and Huang et al.^19^, respectively. To eliminate the redundancy of the dataset and ensure the validity of the experiment, we performed a screening work to remove the redundant protein pairs^46^. Specifically, protein pairs with fewer than fifty residues are completely removed, as they may be just fragments. Furthermore, considering the presence of homologous sequence pairs, those protein pairs with more than 40% sequence identity were also removed. Finally, we retained the remaining 5594 protein pairs to construct a positive PPIs dataset. At the same time, we also constructed a negative dataset using an additional 5594 non-interacting protein pairs, and their subcellular localization was different. Thus, the final *Yeast* dataset in this experiment consisted of 11,188 protein pairs, which contained 50% negative datasets and 50% positive datasets. Analogously, we constructed 8161 protein pairs for *Human* dataset, which included 4262 non-interacting protein pairs and 3899 interacting protein pairs.

### Position-specific scoring matrix

Gribskov et al.^47^ initially introduced a position-specific scoring matrix (PSSM) for the search for distantly related proteins. PSSM is an evolutionary profile based on feature extraction methods that have been successfully used in various fields of bioinformatics. For instance, protein secondary structure prediction^48^, prediction of membrane protein types^49^, prediction of disordered regions^50^, identification of DNA binding proteins^51^, and protein binding site prediction^52^. To integrate the evolutionary information of proteins, we also used PSSM to predict PPIs in this study. The structure of the PSSM can be represented as a matrix with $T$ rows and 20 columns. It can be interpreted as $P = \{ x_{i,j} :i = 1, \ldots ,T,j = 1, \ldots ,20\} .$ Of these, the rows of the matrix are protein residues and the columns refer to native amino acids. We can use the following formula to describe PSSM:

5 $$P = \left[ {\begin{array}{*{20}c} {x_{1,1} } & {x_{1,2} } & \cdots & {x_{1,20} } \\ {x_{2,1} } & {x_{2,2} } & \cdots & {x_{2,20} } \\ \vdots & \vdots & \vdots & \vdots \\ {x_{T,1} } & {x_{T,2} } & \cdots & {x_{T,20} } \\ \end{array} } \right],$$

where $T$ represents the length of the protein sequence, and the element $x_{i,j}$ of PSSM refers to the residue score of the $i{\text{th}}$ residue mutated to the type $j$ amino acid during biological evolution.

In this paper, we employed the Position-Specific Iterated BLAST (PSI-BLAST)^53^ program and the SwissProt database on a local machine to transform each protein sequence into a matrix of score values to further construct experimental datasets to predict PPIs^54^. In the process of running PSI-BLAST, we hope to select highly homologous sequences, and mainly employ these aligned sequences to construct a new scoring matrix. This matrix is called the Position-Specific Scoring Matrix (PSSM), and is weighted according to the kinds of high homology found in the initial hit list. Using this matrix again, we do a blast to pick any new homologous sequences as our scoring schema will change. This process is repeated until no new sequences can be found. PSI-BLAST is more sensitive compared to BLAST, especially in terms of discovering new members of protein families. To generate highly homologous sequences, the important parameter cutoff e-value and the number of iterations of PSI-BLAST were set to 0.001 and 3, respectively, while other parameters were default values. The applications of PSI-BLAST can be publicly accessed at http://blast.ncbi.nlm.nih.gov/Blast.cgi.

### Orthogonal locality preserving projections (OLPP)

Orthogonal locality preserving projections (OLPP) algorithm is an effective manifold learning method. It was used early in the recognition of human faces and was proposed by Deng Cai et al.^55^. This algorithm is extended based on Locality preserving projections (LPP)^56^. Among them, the theoretical knowledge and detailed derivation of the LPP method can be traced back to Ref.^57^. Suppose we give a set of $n$ D-dimensional data $x_{1} ,x_{2} , \ldots ,x_{n}$ through $n$ d-dimensional vectors $y_{1} ,y_{2} , \ldots ,y_{n} ,$ respectively, $D > d.$ The objective function of LPP is formally stated below:

6 $$\min \sum\limits_{ij} {\left\| {y_{i} - y_{j} } \right\|}^{2} S_{ij} ,$$

where $S$ represents a similarity matrix and $y_{i}$ is the one-dimensional representation of $x_{i}$ with a projection vector $w.$ Here, $y_{i} = w^{T} x_{i} .$ According to the minimized objective function, LPP will incur a severe penalty if neighboring points $x_{i}$ and $x_{j}$ are projected far away. One possible way to define the similarity matrix $S$ is as follows:

7 $$S_{ij} = \left\{ {\begin{array}{*{20}c} {{\text{exp}}\left( { - \frac{1}{t}\left\| {x_{i} - x_{j} } \right\|^{2} } \right){,}} & {\left\| {x_{i} - x_{j} } \right\|^{2} < \varepsilon } \\ {0,} & {\text{otherwise,}} \\ \end{array} } \right.$$

where $\varepsilon$ is extremely small, $\varepsilon > 0,$ and the parameter $t$ is seen as a regulator. Here, $\varepsilon$ specifies the radius of the local neighborhood. That is, $\varepsilon$ defines the locality. Thus, the objective function needs to be minimized so that when $x_{i}$ and $x_{j}$ are close, $y_{i}$ and $y_{j}$ are close as well. Finally, the transformation vector $w$ is given by solving the minimum eigenvalue:

8 $$XLX^{T} w = \lambda XDX^{T} w,$$

where $X = \{ x_{1} ,x_{2} , \ldots ,x_{n} \}$ and $\lambda$ represents the eigenvalue and $w$ is the corresponding eigenvector. Here, $L = D - S$ is the Laplacian matrix and $D$ represents a diagonal matrix, $D_{ii} = \sum\nolimits_{j} {S_{ji} } .$ Next, we describe the OLPP algorithm by using the following steps.

1. PCA projection. Principal Components Analysis (PCA) is an effective tool for reducing the dimensionality of multivariate data by using a covariance analysis between factors. PCA projects the input data into an alternate subspace by discarding the portion corresponding to zero eigenvalue. Here, we introduce the *W*_*PCA*_ to represent the transformation matrix of PCA.
2. Contiguity graph construction. OLPP algorithm can construct a K-nearest neighbor (KNN) graph in supervised or unsupervised mode and can also achieve good stability. Let *G* denote a KNN graph with n nodes. The i-th node corresponds to *x*_*i*_ We tend to put an edge between nodes *i* and *j* if *x*_*i*_ and *x*_*j*_ are close, i.e. *x*_*i*_ is among p nearest neighbors of *x*_*j*_. In other words, *x*_*j*_ is among p nearest neighbors of *x*_*i*_. Edges are located between a sample and its K nearest neighbors in an unsupervised setting. Here, K represents a small integer. In general, we use the Euclidean distance metric to measure the closeness between data nodes in a K nearest neighbor graph. In an unsupervised mode, we can get a constructed nearest neighbor graph that approximates the local manifold structure.
3. Selecting the weights. If node *i* and *j* are linked, the weight *W*_*ij*_ is expressed as,
  9 $$W_{ij} { = }e^{{ - \frac{1}{t}\left\| {x_{i} - x_{j} } \right\|^{2} }} ,$$
  where $t$ is a suitable constant. If node $i$ and $j$ are not linked, we have $W_{ij} = 0.$ The weight matrix $W$ of graph $G$ refers to the native structure of the feature space.
4. Computing the orthogonal basis functions. After finding the weight matrix $W,$ we tend to calculate the diagonal matrix $D.$ The diagonal matrix $D$ is defined as the sums of each column element of $W$ (or sums of each row element of $W$ as $W$ is symmetric):
  10 $$D_{ii} = \sum\nolimits_{j} {W_{ji} } .$$

We also calculated the Laplacian matrix $L,$ which is defined as

11 $$L = D - W.$$

Let $\{ o_{1} ,o_{2} ,...,o_{d} \}$ be orthogonal basis vectors, and we define

12 $$A^{(d - 1)} = [o_{1} ,o_{2} , \ldots ,o_{d - 1} ],$$

13 $$B^{(d - 1)} = [A^{(d - 1)} ]^{T} (XDX^{T} )^{ - 1} A^{(d - 1)} .$$

The calculation process of the orthogonal basis vectors $\{ o_{1} ,o_{2} ,...,o_{d} \}$ can be expressed as follows

- Compute $o_{1}$ as the eigenvector of $(XDX^{T} )^{ - 1} XLX^{T}$ associated with the smallest eigenvalue.
- Compute $o_{d}$ as the eigenvector of
  14 $$M^{(d)} = \{ I - (XDX^{T} )^{ - 1} A^{(d - 1)} [B^{(d - 1)} ]^{ - 1} [A^{(d - 1)} ]^{T} \} \cdot (XDX^{T} )^{ - 1} XLX^{T}$$
  related to the minimum eigenvalue of $M^{(d)} .$

5. OLPP embedding. Let $W_{OLPP} = [o_{1} ,o_{2} , \ldots ,o_{s} ],$ the embedding is defined as,
  15 $$x \to y = W^{T} x,$$
  16 $$W = W_{PCA} W_{OLPP} ,$$
  where $y$ is a s-dimensional vector and $W$ is the transformation matrix.

### Rotation forest

In recent years, many ensemble algorithms have been rapidly developed in the field of machine learning, mainly because the ensemble learning classification method can greatly improve the prediction accuracy of classification results. Among them, ensemble classifier built using ensemble machine learning algorithms, such as boosting and bagging methods, usually have much better prediction accuracy than using only a single classifier. In this paper, we use the Rotation Forest (RoF) classifier to perform the classification task of protein–protein interactions. Rotation forest is an ensemble classifier combining decision tree algorithm and principal component analysis theory, which was proposed by Rodriguez et al.^58^. The main idea of the RoF classifier is to improve the diversity and prediction accuracy of the base classifiers by using a transformation approach to perform feature extractions for each classifier^59^. In addition, each decision tree is individually trained and embedded in a rotated feature space utilizing a new dataset in the transformed feature space by the original dataset^60^. Other research literature suggests that the RoF algorithm can achieve better prediction accuracy in classification problems when compared to other ensemble methods^61,62^.

Assuming $X$ be the original training dataset and we can represent it with a matrix of $N \times n.$ Here, $N$ denotes the number of training samples and $n$ denotes the number of features. The corresponding feature set and class label can be represented as $S$ and $Y,$ respectively, where $Y = (y_{1} ,y_{2} , \ldots ,y_{n} )^{T} .$ Let $L$ be the total number of decision tree classifiers present in the RoF, where the ith decision tree is $T_{i} (i = 1,2, \ldots ,L).$ More specifically, the feature set $S$ is first randomly divided into $K$ disjoint subsets in the rotation forest model. In each subset, there are $C = \frac{n}{K}$ features. Here, $K$ and $L$ are two user-defined parameters. Next, we can get $S_{ij}$ and $X_{ij} ,$ where $S_{ij}$ is the jth subset of features for the ith decision tree classifier and $X_{ij}$ is the training dataset $X$ for features in $S_{ij} .$ Based on the bootstrap algorithm, we can generate a new nonempty training set $X^{\prime}_{ij} ,$ which is 75% of the original training dataset. Furthermore, a linear transformation method is applied to $X^{\prime}_{ij}$ to generate a coefficient vector, and it can be described as $\{ a_{{_{ij} }}^{(1)} , \ldots ,a_{{_{ij} }}^{{(C_{j} )}} \} ,$ and the size of each $X^{\prime}_{ij}$ is $C \times 1.$ Subsequently, a sparse rotation transformation matrix $G_{i}$ can be constructed, as shown in the following equation:

17 $$G_{i} = \left[ {\begin{array}{*{20}c} {a_{i1}^{(1)} , \ldots ,a_{i1}^{{(C_{1} )}} } & {\{ 0\} } & \cdots & {\{ 0\} } \\ {\{ 0\} } & {a_{i2}^{(1)} , \ldots ,a_{i2}^{{(C_{2} )}} } & \cdots & {\{ 0\} } \\ \vdots & \vdots & \ddots & \vdots \\ {\{ 0\} } & {\{ 0\} } & \cdots & {a_{iK}^{(1)} , \ldots ,a_{iK}^{{(C_{K} )}} } \\ \end{array} } \right].$$

Then, for a given test sample $x,$ the $d_{ij} (xG_{i}^{a} )$ generated by the decision tree classifier $T_{i}$ is used to determine that the sample $x$ belongs to the class $y_{i} .$ In the next step, the average combination method is used for each class $y_{i}$ to calculate the confidence and the formula is as follows:

18 $$m_{j} (x) = \frac{1}{L}\sum\limits_{i = 1}^{L} {d_{ij} (xG_{i}^{a} )} .$$

Accordingly, for a given test sample $x,$ the main purpose is to assign it to the class with the highest confidence. Thus, to determine whether these protein pairs have interactions with each other.
